# Supplementary material for: Developing a simple risk metric for the effect of sport-related concussion and physical pain on mental health
Source: PLoS One. 2023 Oct 13;18(10):e0292751. doi: 10.1371/journal.pone.0292751 (PMC10575528; doi:10.1371/journal.pone.0292751)
Supplement: S1 Appendix — (DOCX) [file pone.0292751.s002.docx]

**Appendices**

| **Appendix A.** Frequencies of predominant sport | | | |
| --- | --- | --- | --- |
| Predominant Sport | *N* | Total sample (%) | Sport sample (%) |
| Rugby | 43 | 29.9 | 61.4 |
| Athletics | 6 | 4.2 | 8.6 |
| Football | 5 | 3.5 | 7.1 |
| Netball | 2 | 1.4 | 2.9 |
| Touch Rugby | 2 | 1.4 | 2.9 |
| Baseball | 1 | .7 | 1.4 |
| Weightlifting | 1 | .7 | 1.4 |
| Handball | 1 | .7 | 1.4 |
| Squash | 1 | .7 | 1.4 |
| Equestrian | 1 | .7 | 1.4 |
| Skiing | 1 | .7 | 1.4 |
| Cycling | 1 | .7 | 1.4 |
| Badminton | 1 | .7 | 1.4 |
| Boxing | 1 | .7 | 1.4 |
| Cricket | 1 | .7 | 1.4 |
| Dance | 1 | .7 | 1.4 |
| Taekwondo | 1 | .7 | 1.4 |
| Total | 70 | 48.6 | 100 |

| **Appendix B.** Table depicting number of SRCs sustained by sport-type and non-sport | | | | | | | | | | | | | |
| --- | --- | --- | --- | --- | --- | --- | --- | --- | --- | --- | --- | --- | --- |
|  | Number of SRCs | | | | | | | | | | | | |
|  | 1 | 2 | 3 | 4 | 5 | 6 | 7 | 8 | 9 | 10 | 11 | 12 | Total |
| **Contact Sport** |  |  |  |  |  |  |  |  |  |  |  |  |  |
| Rugby | 9 | 9 | 6 | 4 | 6 | 4 | - | 1 | - | 1 | 1 | 1 | 156 |
| Football | 2 | - | - | 1 | - | - | - | - | - | - | - | - | 6 |
| Skiing | 1 | - | - | - | - | - | - | - | - | - | - | - | 1 |
| Boxing | 1 | - | - | - | - | - | - | - | - | - | - | - | 1 |
| Taekwondo | - | - | - | - | - | - | - | - | - | - | - | - | 0 |
| Total | 13 | 9 | 6 | 5 | 6 | 4 | - | 1 | - | 1 | 1 | 1 | **164** |
| **Non-Contact Sport** |  |  |  |  |  |  |  |  |  |  |  |  |  |
| Athletics | 1 | - | - | - | - | - | - | - | - | - | - | - | 1 |
| Netball | - | - | - | - | - | - | - | - | - | - | - | - | 0 |
| Squash | - | - | - | - | - | - | - | - | - | - | - | - | 0 |
| Touch Rugby | - | - | 1 | - | - | 1 | - | - | - | - | - | - | 9 |
| Cricket | - | - | - | - | - | - | - | - | - | - | - | - | 0 |
| Equestrian | - | 1 | - | - | - | - | - | - | - | - | - | - | 2 |
| Baseball | - | - | - | - | - | - | - | - | - | 1 | - | - | 10 |
| Weightlifting | - | - | - | - | - | - | - | - | - | - | - | - | 0 |
| Handball | - | 1 | - | - | - | - | - | - | - | - | - | - | 2 |
| Cycling | - | 1 | - | - | - | - | - | - | - | - | - | - | 2 |
| Badminton | - | - | - | - | - | - | - | - | - | - | - | - | 0 |
| Dance | - | - | - | - | - | - | - | - | - | - | - | - | 0 |
| Total | 1 | 3 | 1 | - | - | 1 | - | - | - | 1 | - | - | **26** |
| **Non-sport** | 3 | - | 1 | 1 | 1 | - | - | 1 | - | 1 | - | - | **33** |
| **Total** | 16 | 25 | 15 | 11 | 13 | 10 | - | 3 | - | 5 | 2 | 2 | **223** |
