# Supplementary material for: Developing a simple risk metric for the effect of sport-related concussion and physical pain on mental health
Source: PLoS One. 2023 Oct 13;18(10):e0292751. doi: 10.1371/journal.pone.0292751 (PMC10575528; doi:10.1371/journal.pone.0292751)
Supplement: Attachment — Submitted filename: Responses_and_Amendments.docx [file pone.0292751.s003.docx]

Manuscript ID PONE-D-22-31831 titled “The likelihood of developing depression following sport-related concussion”

**PLOS ONE**

The authors have attempted to address the editor’s suggestions as highlighted below.

| **Reviewer 1’s comments to Authors** |  |  |
| --- | --- | --- |
| **Comment/Recommendation** | **Amendment** | **Location/Line** |
| You address an important topic in sport-related concussion (SRC) research and highlight the utility of your findings in clinical practice. In particular, I appreciate that the discussion section included the complex point of risk vs. reward in participation in sport, as increased risk for depression is associated with SRC while at the same time, participation in athletics and exercise in general is a protective factor against depression. Overall, the revisions primarily address wording within the manuscript, as well as interpretation of previous research and your own data. I make several comments in the manuscript about wording that draws assumptions about previous research (and at times, mis-cited research) or may be belittling towards certain populations. | Thank you for reviewing our manuscript. We appreciate the time and effort you have gone into reading our work and the helpful comments that you have listed below. We have worked our way through these comments and believe it has added much value to our work. | - |
| Lines 60-73: You briefly discuss the findings of biological sex and symptoms of depression, in that females are two times more likely to experience depression. However, your explanation of the reasons behind this finding in previous research is simplistic and you call into question the accuracy of this data. While I agree that men may under-report symptoms, this finding of women being more likely to endorse depression has been replicated across age groups and different cultures, suggesting it is likely not as simple as under-reporting, but rather a complex constellation of factors, including biological factors (e.g., hormones), environmental factors, and stress and esteem. While I understand that a significant level of detail is beyond the scope of this paper, I do think the paper as written negates the complexity of these differences and should be addressed. | Thank you for this comment. We appreciate the time taken to explain your point of view on this paragraph. We agree that we perhaps over-simplified the processes that lead to women more often presenting depressive symptoms. It was our intention to convey our justification for including sex as a factor in our analysis due to men being more likely to die by suicide while women are more likely to be depressed. Hopefully our amended paragraph here conveys that message more clearly. | L.60-72 |
| The use of the phrase “careless attitudes” to explain perceptions to SRC appears to be an over-generalization and assumption of the research cited. In fact, Anderson, et al. discusses concussion non-disclosure, not willingness to play despite concussion risks. Anderson, et al and other studies further demonstrate that willingness to play despite concussion risk is often multifactorial and not necessarily individual “carelessness”, but rather also due to environmental pressures and expectations. | Thanks for this. We agree that the word “careless” is perhaps unfair to athletes and have subsequently removed this word from the sentence. We believe the sentence still conveys the meaning we wanted it to and is grammatically correct. | L.95 |
| Line 113: You write, “Though there may be a popular belief that females are more likely to be depressed than males…”. This is not just a “popular belief”, but is demonstrated across numerous research studies that take into consideration not only personal factors, but biological, psychosocial, and socioeconomic factors as well. | Thank you for pointing this out. We agree and have amended this sentence accordingly. | L.112-114 |
| Lines 138-139: You indicate that 62 participants reported having sustained an SRC, totaling 223 SRC’s, which would mean be an average of 3.6 concussions per respondent endorsing concussion. That’s a lot! How was SRC reported in the survey? Did you ask whether the SRC was diagnosed by a licensed healthcare provider? I wonder if this number is inflated by self-report alone if there was not confirmation of diagnosis. It would be helpful to include more information about what was assessed in SRC history. If only based on self-report without a question about a confirmed diagnosis, then that should be mentioned as a limitation to the study. | SRC was self-reported and no formal diagnoses were obtained. We have added a statement that makes this explicit and added this to the limitations section as well. | L.128-130 |
| Lines 244-247: The link between pain scale endorsements and level of depression is an important finding. However, I greatly question how the interpretation that females may experience depression at lower levels of physical pain due to “catastrophizing and rumination” fits into the current study on SRC. I further read this finding as disparaging toward females, particularly in light that no other explanations are offered about differences in pain interpretation between males and females. In fact, there are studies on athletes specifically that have suggested that males perceive pain in sport more sensitively than women. I strongly suggest editing this paragraph and removing this interpretation. | Thank you for this comment. Firstly, our apologies. It was never our intention to be disparaging towards females, only to highlight the importance of Walker & McKay (2022) finding and how this study compliments those findings. We have therefore removed the comments regarding catastrophising and rumination and added a final sentence making it clearer why it is important to understand sex differences with pain and subsequent mental health in athletes. | L.261-264 |
| Line 255-256: Concussion does not result in structural changes to the brain. It is a pathophysiological process in the brain. The following line is incorrect and should be removed: “This finding could be due to structural brain changes following collision that exacerbate depression symptoms.” Further, the cited study (Cho et al) highlights premorbid differences in structural sizes in association with mood symptoms, not post-injury structural changes. | Thank you for signposting this to us. We agree and have subsequently removed this sentence from this paragraph. | - |
| Previous research has shown that knowledge of concussion does not lend to improvement in disclosure of concussion (Anderson et al, cited in your paper). With this information in mind, how would knowledge of risks for depression and pain change participation in sports, particularly contact sports? I agree that a risk metric is helpful to share, but what evidence supports that a risk metric will result in behavioral change? | Thank you for this comment. We appreciate this concern and understand it. However, we would argue that the knowledge of many behaviours that pose negative consequences do not lend to the improvement of behaviour. For example, we are aware of the dangers of drinking alcohol, smoking, eating fatty foods, but many still engage in those activities. This is similar, and the line of work regarding concussion in sport, in our view, lends its hand to ensuring sportspeople have an informed decision towards their participation in sport. Where we now see lawsuits against governing bodies of contact sports around the world, particularly the UK, USA and Australia, athletes are citing that they did not know the true dangers of the sports they were competing in. Therefore, this simple risk metric will go some way to helping this, and even if it changes just one person’s view on whether they want to participate or not, that is still a useful, valuable contribution. | - |
| What is the best way to share the risk metric? Having a risk metric is great, but how should that be delivered to athletes? The manuscript states numerous times how important a risk metric can be, but how should dispersion of this information be executed to ensure athletes make informed decisions about play? | This is a good comment and consideration. Our ideas for this manuscript, beyond publishing in your journal and hitting an academic audience in that sense is for this information to be available to sports clubs. As an ambassador for “Give A Ruck” a UK based charity whose aim it is to improve the stigma regarding mental health in rugby, I intend to share these findings with this charity and therefore all the rugby clubs associated with the charity and players within those clubs. Beyond this, an advertising campaign could also be useful to disseminate this information. I would also be presenting this at international conferences. | - |
| **Reviewer 2’s comments to Authors** |  |  |
| **Comment/Recommendation** | **Amendment** | **Location/Line** |
| The authors examined factors related to depression in a convenience sample of adults. Contact sport participation, prior concussion, male sex, and greater pain were significant predictors of depression group (high vs. low current symptoms). This is a topic of interest to the journals readership. Strengths of this manuscript include that the authors enumerate their hypothesized associations, the involvement of the research ethics committee, and appropriate informed consent. Substantial limitations to the manuscript need to be addressed, which greatly outweigh strengths. Major criticisms include the authors' failure to recognize the limitations of their methods. I would like to see a CONSORT-type diagram characterizing the sample. I would also like to know that the assumptions for multicollinearity were not violated. The authors imply causality of depression; however this cannot be inferred from these data. And, a major basis for one of their hypothesis (that men are reluctant to self-report depression) cannot be addressed with the self-report depression measure they use. Though not fatal flaws, substantial revisions to the manuscript are needed. I offer the following more specific suggestions as well: | Thank you for reviewing our manuscript. We appreciate the time and effort you have gone into reading our work and the helpful comments that you have listed below. We have worked our way through these comments and believe it has added much value to our work. | - |
| Line 31: This statement should be revised to clarify that conclusions surrounding directionality cannot be assumed from the authors' findings. While predictors might cause depression, it might also be that depressed adults more frequently play contact sports, sustain concussions, belong to a specific sex, or have comorbid pain. | Thank you for this comment. We have now amended this to suggest that SRC and pain are associated with poor mental health. | L.30-31 |
| Lines 50-59: It is unclear how the stated problem (complexity of presenting athletes with depression risk information) and the need for "behaviour change in athletes" is related. Looking to past literature, the authors should quantify the size of the association and amount of increased risk for depression the athlete assumes. | Thanks for this comment. It is impossible to quantify the amount of risk an athlete is willing to take. Instead, what we do know is that many athletes are willing to take the risk of sustaining SRC and experiencing physical pain, and we know this because they are engaging in contact sports where they are prevalent risks. If we were to ask any athlete of whether they understand the risk of pain and SRC they are likely to say that they do, but still compete regardless. So, our assumption is that while some may know the true risk, many may not, and therefore our paper is presenting a simple risk metric that tells athletes if you are:  - Female (3x)  - Have had concussion (57x)  - Play contact sport (71x)  - In pain (1.4x for every score of pain)  …more likely to develop depression.  With this information, we hope that athletes that claim to know the risk but may not have a more informed decision of taking part in contact sports where these outcomes and consequences are likely. | - |
| Lines 132-133: The authors should note the number of recent concussion exclusions for this sample, or report if the number screened ineligible is not known. | Concussions were self-reported in this sample, and number of concussions was the same, they just reported how many the had had. This is why there is a wide range of number of concussions (*Range,* 1-12, *M* = 3.60, *SD ­*= 2.80) as highlighted in the participants sub-section. | L.128-130 |
| Line 146: What was the basis for using an effect size of 0.5 in power analyses? | Thanks for this comment. In our line of work we tend to use a large effect size in power analysis due to the time differences between variables. For example, SRC history occurred between 1 and 48 months prior to participation and we asked participants to report on their pain in the past week. Therefore we expect a large effect size due to these time differences. We have added a note in this here to explain this. Thanks for bringing it to our attention. | L.146-148 |
| Line 152: Please note that the acronym's D represents depression scale in "Centre for Epidemiological Studies (CESD..." | Thank you for this, an oversight on our part. | L.154 |
| Lines 159-160: Given that a higher depression score indicates more severe depression symptoms, the authors should provide rationale for dividing their outcome measure into two groups rather than examining continuously. | Thanks for this comment. You are quite right that we can use this tool in two separate ways, a continuous way whereby higher scores = worse depression or we can use it in a categorical way where Husaini et al. (1980) suggest a score of 16 or above is classified as depressed and below this value is not depressed. Therefore, the use of this is usually driven by the analysis technique adopted. As we used logistic regression, and one of the assumptions of this analysis technique is that the dependent variable is binary, this is why we decided to divide the depression measures into depressed/non-depressed using this cut-off score of 16 on the CESD. | - |
| Lines 191-194: I am suspicious that this association between contact sports and concussion suggests may violate an underlying assumption of regression, multicollinearity. The authors should discuss how statistical assumptions were checked. | Thank you for this comment. We have added a sub-section to the method section titled “assumptions” which hopefully answers your questions here. We have also added a table of correlations of independent variables to aid in your interpretation. | L.187-197 |
| Lines 226-237: That men potentially under-report depression symptoms is a red herring the present study does not address. The authors propose that males may have more severe depression than captured in prior study. However, they lack support for sex as a biological factor influencing depression symptomatology. | Thanks for this comment. Given that we amended a paragraph in the introduction to include common explanations of higher prevalence rates of depression in women, we included this here too, so not to solely conclude that underreporting in males is the main reason for this. However, we maintain that underreporting in males particularly male athletes is a viable contributor to what we find here regarding women being 3x more likely to develop depression. Therefore, we have rephrased this paragraph but maintained the suggestion of underreporting in males and do not see this as a red herring or outside the scope of this paper. | L.239-246 |
