# Supplementary material for: Developing a simple risk metric for the effect of sport-related concussion and physical pain on mental health
Source: PLoS One. 2023 Oct 13;18(10):e0292751. doi: 10.1371/journal.pone.0292751 (PMC10575528; doi:10.1371/journal.pone.0292751)
Supplement: Attachment — Submitted filename: Responses_and_Amendments.docx [file pone.0292751.s004.docx]

Manuscript ID PONE-D-22-31831 titled “Contact sports and sport-related concussion are linked to elevated depressive symptoms in athletes and non-athletes”

**PLOS ONE**

The authors have attempted to address the editor’s suggestions as highlighted below.

| **Reviewer 2’s comments to Authors** |  |  |
| --- | --- | --- |
| **Comment/Recommendation** | **Amendment** | **Location/Line** |
| I have had the opportunity to review the revised manuscript, responses, and other review. I appreciate the authors' substantial corrections to address major limitations identified during review. Overall, this paper still conveys findings in a misleading way that requires major revisions. I have included my additional considerations below: | Thank you once again for taking the time to read our work and provide excellent comments that we feel have improved the value of our manuscript once more. | - |
| The title does not convey that the study included >50% non-sport participants and should be modified. | Thank you for this comment. We have amended the title now to make this explicit to the reader. | L. 5-6 |
| Abstract Line 31: More grammatically correct would be "associated with poor mental health." | Thank you for this. We have checked the grammar here and have changed the sentence accordingly. | L. 29-31 |
| Introduction Lines 57-59: Revise or omit the commentary regarding behavior change as it is out of scope and contradictory to the authors' responses noting that "we would argue that the knowledge of many behaviours that pose negative consequences do not lend to the improvement of behaviour." | Thank you for this comment, we have amended this sentence. | L. 57 |
| Line 73: As this was not an exclusively athlete sample, it would be relevant to additionally include here prior literature on the risk of pain and depression in non-sportspeople. | Thanks for this comment. We have now added more detail here, separating it into two paragraphs to make the argument that pain impacts both athletes and non-athletes, but that the pain that athletes are experiencing is more controllable, given their voluntary participation. This paper is aimed at providing athletes with the information to make an informed decision of their participation. Physical pain may be viewed as “part of the game” and they may not be aware that it is detrimental to their mental health, hence this paper. | L. 72-96 |
| Methods Line 129: It would be helpful to include more information on concussion history. Include the title of the survey (if one was used) used or specific questions asked. It is unclear if participants's responses were exclusive to concussions previously sustained during sport. | Thank you for this. We have added a little bit more information regarding the type of questions used about concussion. We also mentioned in the *measures* section that concussion questions were asked among the General Information Questionnaire. | L. 139-41 |
| Line 197: In table 1, was the correlation performed on dummy variables for categorical factors e.g. sex? What is the full citation for Senaviratna & Cooray, 2019? | Apologies for the oversight in omitting the citation from the reference list. This has now been added in. Yes, dummy coding was used for both regression and correlations. | - |
| Results Lines 205-206: Again, it is unclear if this variable reflects only SRC. Did the 8 non-sport participants with prior concussion sustain their injuries in sport only? | Thanks for this comment. Yes, these would have been SRC’s sustained from sport despite them no longer being part of sport and in the non-sport group. Hopefully the way we have addressed this in the *participants* section now makes this clear. | - |
| Line 208: The mean depression score is greatest for the non-sport group, counter to the findings of the binary regression. What are the author's interpretation of this? | There is a discrepancy here due to descriptive statistics utilising raw continuous scores as opposed to the binary regression using (depressed/non-depressed) categories. Therefore, we have included the % of each group that were in the depressed categorisation in table 1. to inform the reader the proportion of each group were in said category for the regression. We can now see that 63% of the non-sport group were in the depressed group while only 47% and 39% of the non-contact sport and contact sport groups respectively were. This strengthens our argument, as despite there being more ”depressed” people in the non-sport group, our regression finds that non-contact sport and contact sport, SRC and pain (that are prevalent in both) to be associated with a higher likelihood of being depressed. | Table 1. |
| Line 227: The extremely large confidence interval for the contact sport OR (6.37 - 1000) should be addressed in the discussion section. | Good spot, this is very important in the interpretation of findings. We have added a few sentences in the discussion section that outlines how this should be interpreted, in that even taking the lowest confidence interval does not alter the message we are conveying and that anything above this figure only makes our argument stronger. | L. 298-305 |
| Discussion Lines 233-234 & 282-284: Given the overlapping confidence intervals for non-contact and contact sport, it may be that these groups have similar degree of depression risk. | Thank you for this comment. We have added a paragraph explaining this and how this is not an issue for the meaning of the paper but that it should be noted as it could be that there is not so much of a difference between sport-type, but just sport v non-sport generally. | L. 326-334 |
| Line 297: What is meant by misclassifications, as in what processes is suspected as underlying? | Here we meant that it is unlikely that all participants have always been part of the group that they were when they took part in the study. It is unlikely that those in the non-sport group have never played sport before and that the non-contact sport group have never played contact sports and so on. Therefore, the finding that those that take part in no sport at all is better for mental health is non-sensical given what we know about the positive influences of physical activity. Instead, we suggest that this could be due to misclassifications of group in that individuals may once have been part of other groups. We then go on to provide examples of evidence of this (11% of the no sport group had sustained SRC – by definition they must have once played sport to have sustained sport-related concussion). | - |
| Lines 366-368: It is unclear what evidence supports that the non-sport group has sustained "painful repetitive strain injuries...and withdrawn from that sport due to its effects." | Thanks for this comment. We agree that it may be a bit of a stretch and have omitted this and replaced it with a different sentence. This reiterates that part of the non-sport group had sustained SRC and were therefore once sportspeople and that this could influence the results. | L. 394-395 |
