# Supplementary material for: Developing a simple risk metric for the effect of sport-related concussion and physical pain on mental health
Source: PLoS One. 2023 Oct 13;18(10):e0292751. doi: 10.1371/journal.pone.0292751 (PMC10575528; doi:10.1371/journal.pone.0292751)
Supplement: Attachment — Submitted filename: Responses_and_Amendments.docx [file pone.0292751.s005.docx]

Manuscript ID PONE-D-22-31831 titled “Developing a simple risk metric for sport-related concussion and physical pain on mental health”

**PLOS ONE**

The authors have attempted to address the editor’s suggestions as highlighted below.

| **Reviewer 2’s comments to Authors** |  |  |
| --- | --- | --- |
| **Comment/Recommendation** | **Amendment** | **Location/Line** |
| The authors have adequately addressed the majority of my comments from my latest review. However, there appears to be a major interpretive error in the regression results. Looking at the Table 3 column for B, depression risk is increased (positive coefficient beta) for sex, pain, and SRC but decreased (negative coefficient beta) for sport type. Thus the OR for a beta of -1.48 is about 0.23 (not 4.37, which is the OR for a beta of +1.48). Similarly, the OR for beta of -4.28 is about 0.01 (not 71.43, which is the OR for a beta of +4.28). This would align with Table 1's findings that the non-sport group had the highest rate of depression. Revision would be needed to incorporate this interpretation, which is opposite to the author's currently stated interpretation of findings. I strongly recommend consultation with an individual expert in statistics to avoid further oversights. | Thank you for this comment. We have taken this on board and had another look at the statistics. We appreciate your efforts to explain this, however it is Exp(B) that needs inverting as the odds ratios rather than B. B as you rightly point out provides us an indication of the direction of the relationship whereas Exp(B) is the OR. Therefore, our calculations were correct the first time around, in that the OR for Non-contact sport is 4.37 and for contact sport is 71.43, but that participants of non-contact sports are 4.37 times **less** likely to be depressed than non-sportspeople and those that took part in contact sports were 71.43 times **less** likely to be depressed than sportspeople. We have amended the table now to present the original values that are under 1 (as this suggests the direction too, if you are less than 1x likely to experience x because of y then you are less likely to experience it.) We have also included the inverted values next to these to aid the reader in interpreting this so they are aware of the degree of how much less likely these individuals were to experiencing depression.  We have amended our interpretation in the discussion accordingly, as well as necessary amendments to the abstract and results sections and title. | - |
| Lines 139-140: I presume "how many (free response in months)" should be "how long ago". | Thank you for spotting this oversight. This was two separate questions, one to ask how many concussions participants had experienced in total and how many months had passed since the last one had occurred. Both were free-text responses. We hope our amendment clears this up. | L. 138-140 |
| Line 164: Please spell out the first instance of Numerical Rating Scale (NRS) here. | Thank you for spotting this oversight, we have now amended this. | L. 164 |
